# Does β-Hydroxy-β-Methylbutyrate Have Any Potential to Support the Treatment of Duchenne Muscular Dystrophy in Humans and Animals?

**DOI:** 10.3390/biomedicines11082329

**Published:** 2023-08-21

**Authors:** Abdolvahab Ebrahimpour Gorji, Piotr Ostaszewski, Kaja Urbańska, Tomasz Sadkowski

**Affiliations:** 1 Department of Physiological Sciences, Institute of Veterinary Medicine, Warsaw University of Life Sciences, 02-776 Warsaw, Poland; abdolvahab_ebrahimpourgorji@sggw.edu.pl (A.E.G.); piotr_ostaszewski@sggw.edu.pl (P.O.); 2 Department of Morphological Sciences, Institute of Veterinary Medicine, Warsaw University of Life Sciences, 02-776 Warsaw, Poland; kaja_urbanska@sggw.edu.pl

**Keywords:** DMD, HMB, genes, pathways, biological process

## Abstract

Skeletal muscle is the protein reservoir of our body and an important regulator of glucose and lipid homeostasis. The dystrophin gene is the largest gene and has a key role in skeletal muscle construction and function. Mutations in the dystrophin gene cause Duchenne and Becker muscular dystrophy in humans, mice, dogs, and cats. Duchenne muscular dystrophy (DMD) is an X-linked neuromuscular condition causing progressive muscle weakness and premature death. β-hydroxy β-methylbutyrate (HMB) prevents deleterious muscle responses under pathological conditions, including tumor and chronic steroid therapy-related muscle losses. The use of HMB as a dietary supplement allows for increasing lean weight gain; has a positive immunostimulatory effect; is associated with decreased mortality; and attenuates sarcopenia in elderly animals and individuals. This study aimed to identify some genes, metabolic pathways, and biological processes which are common for DMD and HMB based on existing literature and then discuss the consequences of that interaction.

## 1. Introduction

Over the course of three decades, the effects of β-hydroxy-β-methylbutyrate (HMB), a metabolite of leucine, on skeletal muscle mass and strength have been extensively studied [1]. HMB forms endogenously through leucine’s reversible transamination to α-ketoisocaproic acid (KIC), catalyzed by branched-chain amino acid (BCAA) transaminase, primarily situated within skeletal muscle. The body’s production of KIC accounts for roughly 15% of the turnover rate of leucine, an essential amino acid crucial for various physiological processes. In comparison, the production of HMB, a metabolite derived from KIC, constitutes less than 1% of KIC turnover and a mere 0.1% of leucine turnover. Notably, the overall synthesis of HMB is estimated at approximately 4 μmol/kg of lean body mass per day. These metabolic proportions emphasize the relatively small but impactful role of HMB in the broader turnover of leucine and its subsequent metabolites [2]. HMB is believed to have several mechanisms of action, including the stimulation of the mammalian target of rapamycin (mTOR), which results in increased protein synthesis [3]. Moreover, HMB exerts an effect on protein degradation by inhibiting the ubiquitin-proteasome proteolytic pathway in muscle cells [4]. Immobilization and catabolic conditions induce ubiquitin, leading to proteasome expression through nuclear factor kappa B (NF-κB), thereby promoting muscle wasting. NF-κB activity can lead to the expression of proteasome through ubiquitin, promoting muscle wasting. However, HMB has been shown to reduce muscle loss by inhibiting the activity of NF-κB [5]. The efficacy of HMB supplementation has been studied in a variety of clinical conditions characterized by loss of skeletal muscle mass and weakness, including HIV (human immunodeficiency virus) [6], critical illness [7], and aging [8]. Moreover, prior research has demonstrated that the utilization of HMB supplementation in individuals suffering from cancer cachexia leads to a deceleration in skeletal muscle breakdown and the promotion of protein synthesis [4,9]. Additionally, some research works indicated that the MAP kinase and PI3K/AKT signaling pathways are implicated in these advantageous impacts of HMB on skeletal muscle [10,11]. Nevertheless, the precise molecular mechanisms involved in these processes have yet to be wholly ascertained and require further inquiry.

Muscular dystrophies are a heterogeneous cluster of hereditary disorders that affect skeletal muscles and are primarily characterized by the progression of muscle weakness and degeneration. This, in turn, leads to a reduced lifespan of the affected individuals. The prevalence of muscular dystrophy in the general population can vary significantly depending on the specific type of muscular dystrophy and geographical region. A systematic literature review, encompassing studies classified as having a low risk of bias, reported a total combined prevalence ranging between 19.8 and 25.1 cases per 100,000 person/year for all types of muscular dystrophies [12]. Among children, the most common muscular dystrophy is Duchenne muscular dystrophy (DMD), which is an X-linked inherited disease [13] caused by the absence of the protein dystrophin, which is encoded by the DMD gene. DMD prevalence and birth prevalence estimates are variable throughout the literature, ranging from 0.9 to 16.8 per 100,000 males from 1.5 to 28.2 per 100,000 live male births, respectively [14]. The mutation in the DMD gene disrupts the synthesis of this protein, which plays a crucial role in muscle contraction, and its deficiency results in structural and signaling malfunctions, leading to the gradual breakdown of muscular fibers and chronic inflammation, which is a significant feature of this disease [15]. During the process of inflammation, neutrophils that are present in the bloodstream move toward the affected area, followed by the monocytes and lymphocytes [16]. These cells release cytokines such as tumor necrosis factor-alpha (*TNF-α*), interleukin 1 (*IL1*), and *IL6*, which are classified as proinflammatory; and *IL10*, which is classified as an anti-inflammatory cytokine. The transcription factor NF-κB regulates the release of all these cytokines. This factor may be involved in the pathogenesis of DMD. Skeletal muscle inflammation is regulated by cytokine homeostasis [17], However, in boys with DMD, there is an imbalance in the release of *TNF-α*, *IL1*, and *IL6*, which are increased, and *IL10*, which is decreased, as compared to healthy individuals [18], indicating an imbalance in the pro- and anti-inflammatory activity. The chronic inflammation described in DMD could be associated with decreased muscle function, repetitive cycles of degeneration/regeneration, and muscle atrophy [19,20,21]. Children due to their muscle weakness usually are forced to use a wheelchair by the age of 12 and die early in their third decade of life from respiratory or cardiac failure [22].

The recommended medical treatment for DMD involves the administration of glucocorticoids, which act by diminishing inflammation through the reduction of inflammatory cell counts in impaired muscle fibers [23]. Nevertheless, glucocorticoids carry the potential for significant deleterious side effects. Thus, alternative therapeutic approaches aimed at mitigating the inflammatory process induced by muscle fiber impairment in DMD patients are required to diminish subsequent muscle damage. Payne et al. (2006) [24] studied the efficiency of HMB in a mouse model of DMD and found that HMB significantly decreases plasma creatine kinase (CK, DMD enzymatic indicator of muscle damage) [24]. Since 1994, HMB holds U.S. Patent 5,348,979 “Methods of Promoting Nitrogen Retention in Humans”. There are also international patents, mostly European, that correspond to U.S. patents. The use of HMB in Poland as a dietary supplement in humans was approved by the Polish Authorities in 1996. Soon after, HMB was offered for young boys with genetically diagnosed DMD. Beneficial effects of HMB administration were observed from the very beginning of HMB treatment (unpublished). Parents of sick kids reported an improvement in their children’s motion, slower progression of a disease, better tolerance for exercise, and improved walking ability. In all HMB-treated children, there was a significant decrease in the activity of serum CK when compared with patients not treated with HMB. The oldest patient who is still successfully taking HMB (since the first symptoms of the disease) is now 32 years old. To date, more than a dozen individuals with DMD have been taking HMB supplements, with some of them having taken it for more than 20 years now (unpublished).

In this study, Pathway Studio Web Mammalian (V. 12.5.0.2, 2022) software was used to identify interactions between molecular factors common for HMB and DMD. There are over 24 million PubMed abstracts and 3·5 million Elsevier full-text papers in this database. Pathway Studio was useful for the searching, examining, and visualization of relevant relationships among different kinds of molecular factors such as genes, proteins, and metabolites, based on information on their interactions present in the aforementioned database [25].

To our knowledge, this paper presents the first attempt to investigate the potential effect of HMB on DMD. Using an extensive literature review and in silico analysis, the aim of this article is to demonstrate the potential of HMB in ameliorating the negative consequences associated with DMD and its therapeutic agents by the identification of some genes and pathways that are common for HMB and DMD, and analysis of the consequences of such interaction.

## 2. Common Pathways

**Figure 1:**
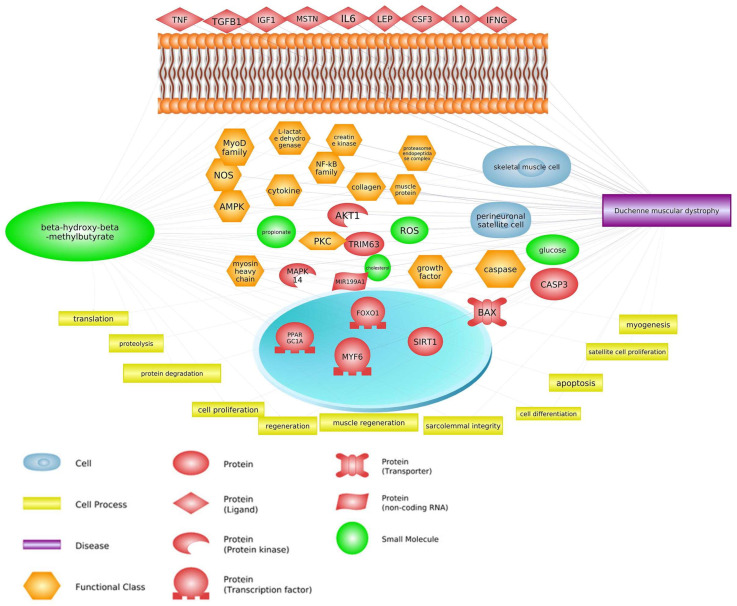
Relevance network of genes, transcription factors, small molecules, functional classes, miRNA, and biological processes that are shared between DMD and HMB (based on Pathway Studio Web Mammalian). Tumor Necrosis Factor (TNF), Transforming Growth Factor Beta 1 (TGFB1), Insulin-Like Growth Factor 1 (IGF1), Myostatin (MSTN), AKT Serine/Threonine Kinase 1 (AKT1), Leptin (LEP), Interleukin 6 (IL6), Caspase 3 (CASP3), Mitogen-Activated Protein Kinase (MAPK14), Myogenic Differentiation (MyoD), Nitric Oxide Synthase (nNOS), Nuclear Factor Kappa B Subunit (NF-κB), Lactate Dehydrogenase (LDH), Interleukin 10 (IL10), Colony Stimulating Factor 3 (CSF3), Tripartite Motif Containing 63 (TRIM63), Interferon Gamma (IFNG), Forkhead Box O1 (FOXO1), Myogenic Factor 6 (MYF6), Caspase 3 (CASP3), BCL2 Associated X, Apoptosis Regulator (BAX), PPARG Coactivator 1 Alpha (PPARGC1A), Reactive Oxygen Species (ROS), Protein Kinase C (PKC), AMP-activated Protein Kinase (AMPK).

**Figure 2:**
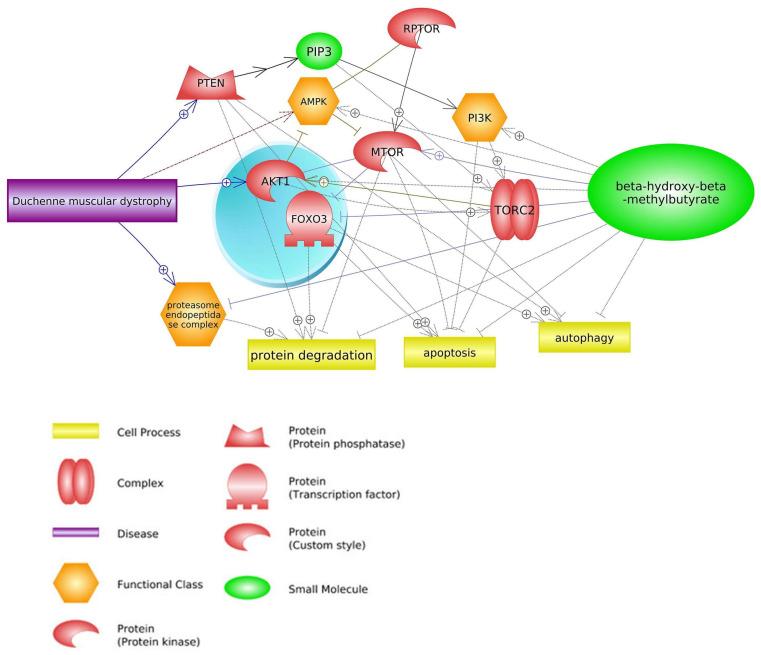
Genes common for DMD and HMB—mTOR signaling pathways (based on Pathway Studio Web Mammalian [47,48,49,50,51,52,53,54]). Phosphatase and Tensin Homolog (PTEN), Regulatory Associated Protein of mTOR Complex 1 (RPTOR), CREB Regulated Transcription Coactivator 2 (TORC2), AKT Serine/Threonine Kinase 1 (AKT1), Forkhead Box O3 (FOXO3), Phosphatidylinositol (3)-trisphosphate (PIP3), AMP-activated Protein Kinase (AMPK), Phosphatidylinositol 3-kinase (PI3K), Mechanistic Target of Rapamycin (mTOR). The “+” symbol at the end of the arrow corresponds to stimulation, the “┤” symbol corresponds to inhibition.

**Figure 3:**
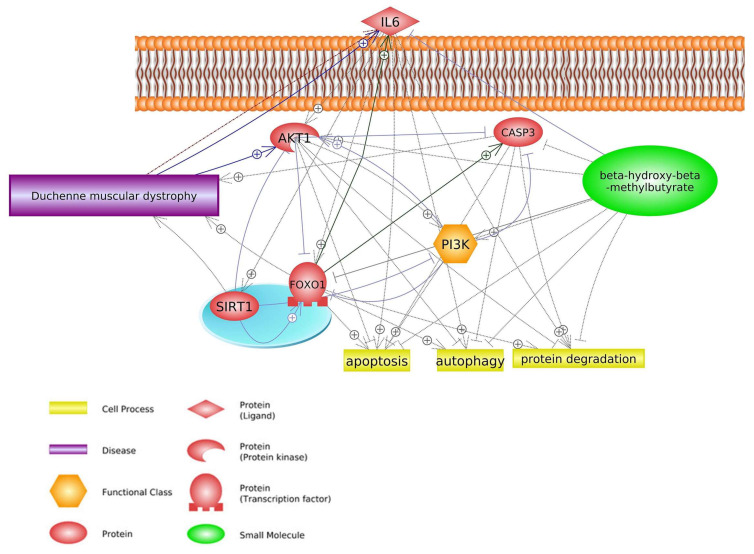
Genes common for DMD and HMB—FOXO1 signaling pathways (based on Pathway Studio Web Mammalian [17,58,63,66,67,68,69]). AKT Serine/Threonine Kinase 1 (AKT1), Interleukin 6 (IL6), Caspase 3 (CASP3), Forkhead Box O1 (FOXO1), Phosphatidylinositol 3-kinase (PI3K), Sirtuin 1 (SIRT1). The “+” symbol at the end of the arrow corresponds to stimulation, the “┤” symbol corresponds to inhibition.

**Figure 4:**
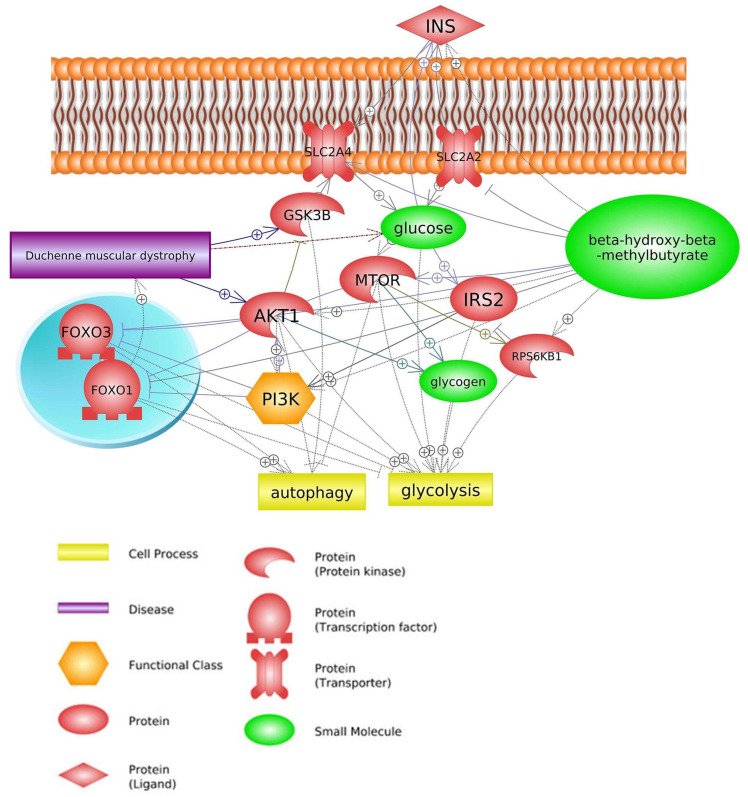
Genes common for DMD and HMB—insulin signaling pathway (based on Pathway Studio Web Mammalian [3,48,49,52,54,66,79,80]). Insulin (INS), Solute Carrier Family 2 Member 4 (SLC2A4), Solute Carrier Family 2 Member 2 (SLC2A2), Glycogen Synthase Kinase 3 Beta (GSK3B), Ribosomal Protein S6 Kinase B1(RPS6KB1), Insulin Receptor Substrate 2 (IRS2), AKT Serine/Threonine Kinase 1 (AKT1), Forkhead Box O1 (FOXO1), Forkhead Box O3 (FOXO3), Mechanistic Target of Rapamycin (mTOR), Phosphatidylinositol 3-kinase (PI3K). The “+” symbol at the end of the arrow corresponds to stimulation, the “┤” symbol corresponds to inhibition.

Pathway Studio Web Mammalian (V. 12.5.0.2, 2022) software analysis allowed for the identification of genes involved in both the course of DMD and the mechanism of action of HMB (keywords: Duchenne muscular dystrophy, β-hydroxy-β-methylbutyrate, and processes listed in Figure 1, Figure 2, Figure 3 and Figure 4). We suppose that they may be linked to the mechanism of positive action of HMB in individuals with DMD. Based on the common genes between muscular dystrophy and the leucine metabolite, we found pathways that affect both DMD and HMB metabolism. In this part of our paper, the three top pathways and their impact on DMD and HMB are discussed (Figure 1).

### 2.1. mTOR Signaling Pathway

The regulation of cellular growth in response to a variety of stimuli, including energy, nutrients, and growth factors, is governed by the mTOR pathway. This pathway is primarily managed by the mammalian target of the rapamycin protein, which is classified as a member of the phosphoinositide 3-kinase-related protein kinase (PIKKs) family. mTOR assembles into two complexes with distinct inputs and downstream effects. mTOR complex 1 (*mTORC1*) interacts with a novel protein named RAPTOR (regulatory associated protein of mTOR) that regulates cell growth by promoting translation, ribosome biogenesis, and autophagy. mTORC2 responds primarily to growth factors, promoting cell-cycle entry, cell survival, actin cytoskeleton polarization, and anabolic output. mTORC2 is regulated by insulin, growth factors, plasma factors, and the number of nutrients [26,27,28,29].

One of the main factors that has a direct effect on mTOR signaling pathways is HMB supplementation, which leads to the inhibition of protein degradation, apoptosis, and autophagy [30,31,32]. The findings of Baier et al. (2009) [33], Stout et al. (2015) [34], and Ellis et al. (2019) [35] may be attributed to the anabolic properties of HMB, which activates the protein rapamycin in humans. This pathway activation leads to an increase in mRNA translation and phosphorylation of *4EBP-1* and *p70S6k*, which are mTOR effectors. This, in turn, results in the synthesis of myofibrillar protein and a subsequent increase in lean mass [36]. The third mechanism of HMB action that leads to an increase in mTOR activity depends on the elevated expression of insulin-like growth factor-1 (*IGF1*) and growth hormone (*GH*) and leads to an increase in the differentiation and proliferation of satellite muscle cells [11,37]. In addition, HMB can stimulate mTOR activity by the impact on *AKT* serine/threonine kinase 1 (*AKT1*) expression. Overexpression of *AKT1* by HMB supplementation leads to an increase in mTORC1 [38,39] and mTORC2 activities [40,41,42], as well as concomitant inhibition of *AMPK* expression (one of the DMD biomarkers) [43,44]. Moreover, HMB in this pathway inhibits proteasome endopeptidase complex activity that leads to attenuation of protein degradation during DMD [45,46] (Figure 2). Activation of mTOR complexes leads to inhibiting DMD side effects such as apoptosis [29] and protein degradation [28].

### 2.2. FOXO1 Signaling Pathway

The FOXO family of proteins, specifically Foxo1, are nuclear transcription factors that have a significant impact on the regulation of overall energy metabolism in the body. Their presence is ubiquitous across tissues, and they are involved in various processes that control cell proliferation and differentiation. To maintain glucose homeostasis, the production and uptake of glucose in peripheral tissues must be adjusted secretion to insulin. During periods of fasting, the liver is primarily responsible for maintaining glucose levels, and Foxo1 is instrumental in promoting the expression of gluconeogenic enzymes. FOXO1 indirectly regulates the formation of adipose tissue and skeletal muscles, particularly in the presence of metabolic dysfunction or insulin resistance. These proteins hold potential as molecules that bridge the gap between longevity and tumor suppression [55]. FOXO proteins serve as key substrates of the protein kinase AKT when cells are stimulated by growth factors or insulin. The presence of these factors prompts FOXO proteins to move from the nucleus to the cytoplasm, where they are ultimately degraded via the ubiquitin-proteasome pathway. In the absence of growth factors, FOXO proteins translocate to the nucleus and elevate cell cycle arrest, stress resistance, or apoptosis [56].

During muscle inflammation that accompanies DMD, an increase in NF-κB signaling stimulates leukocytes to release *TNF-α* and proinflammatory cytokines *IL1* and *IL6* [57]. *NF-κB* plays a central role in inflammatory reactions and is the primary activator of cytokines. In contrast, HMB suppresses upregulated *IL6* production in response to *TNF-α* stimulation in cancer cell lines in vitro through control of the ubiquitin-proteasome pathway without inducing apoptosis [58]. HMB can inhibit some genes that lead to the progression of DMD or upregulation of this disease, for example, *IL6*, a biomarker of DMD [17], is overexpressed in DMD patients compared to healthy individuals [17,59,60]. In contrast, HMB suppresses the expression of *IL6* in DMD disease, and consequently, apoptosis of muscular cells and DMD side effects will decline [58]. It should be noted that *IL6* in the Foxo1 signaling pathway enhances Foxo1 [61] and Sirtuin 1 (*SIRT1*) [62] expression, and these factors have positive effects on DMD. Another protein that is inhibited by HMB is caspase 3 (*CASP3*) [10]. This protein has not only a positive effect on DMD [63] but also enhances some biological processes such as apoptosis and protein degradation [63,64,65] (Figure 3).

HMB also attenuated the increased protein degradation by *TNF-α* and angiotensin II by inhibiting the formation of reactive oxygen species [10]. Noh et al. (2014) [66] the study discovered that the introduction of HMB supplements led to a decrease in the nuclear translocation of Foxo1. This suggests that further investigation is necessary to determine whether the effects of HMB are associated with the PI3/AKT/mTOR pathway or other signals connected to Foxo. Additionally, since HMB supplementation did not affect serum insulin, which is a crucial signal for the induction of PI3K/AKT, the authors propose that HMB directly influences PI3K/AKT signaling. These results demonstrate that the use of HMB supplements significantly reduces muscle proteolysis through the inhibition of *Foxo1* translocation and the down-regulation of MuRF1, resulting in the inhibition of MyHC degradation.

### 2.3. Insulin Signaling Pathway

Insulin is a highly effective anabolic factor that induces cellular growth and differentiation, while also facilitating the storage of substrates in the liver, muscle, and fat. This occurs through insulin’s stimulation of lipogenesis, glycogen and protein synthesis, and inhibition of lipolysis, glycogenolysis, and protein breakdown [70,71,72]. Insulin also supports glucose transport in muscle and fat cells by promoting the translocation of glucose transporter GLUT4 from intracellular sites to the cell surface. Of the insulin-dependent glucose disposal, roughly 75% occurs in skeletal muscle, while adipose tissue accounts for a relatively small fraction. Additionally, insulin activates mTOR, a member of the PI3K protein family that primarily functions as a serine kinase. The activation of this protein kinase involves PI3K activation, although another signal may also be necessary. The mammalian translation machinery can be regulated by mTOR through direct phosphorylation that activates p70 ribosomal S6 kinase and the initiation factor 4E for the eukaryotic translation inhibitor, PHAS1 or 4E-binding protein 1. As a result of p70’s activation of ribosome biosynthesis by phosphorylating the ribosomal S6 protein, mRNA translation is increased [73]. The release of insulin from the pancreas leads to glucose uptake in peripheral tissues, except the liver, and switches the body from catabolism to anabolism. Insulin inhibits glycogenolysis and gluconeogenesis in the liver while stimulating glycogen accumulation through a coordinated increase in glucose transport and glycogen synthesis [74].

The processes of glycolysis and glycogenolysis are suppressed in DMD patients, leading to a significant decrease in glucose levels, the main energy substrate. This energy deficit is likely the cause of the observed muscular weakness in these individuals [75]. The absence of dystrophin protein in DMD patients results in abnormal glucose transport mechanisms via multicomponent glycoprotein complexes, which may contribute to the reduction in glucose concentration in dystrophic muscle. The phenotype in dogs with golden retriever muscular dystrophy (GRMD) is more severe than in mdx mice, and these dogs, which lack dystrophin, may be a better model for pre-clinical studies to predict outcomes in human DMD.

Dogs with GRMD exhibit a metabolic crisis in their dystrophic muscles, characterized by a reduction in glycolytic enzymes, abnormal and decreased mitochondria, and dysregulated AMP-activated protein kinase (AMPK) expression. The primary glucose transporter, GLUT4, is abnormally located in subcellular aggregates in muscular dystrophy. The translocation of GLUT4 is controlled by insulin and muscle contraction, both of which function through separate molecular pathways to enhance the cytoplasmic vesicle translocation to the cell membrane. Interestingly, in GRMD dogs, GLUT4 was found to be localized to the sarcolemmal membrane, which resulted in the rapid clearance of blood glucose and increased muscle glucose uptake. Collectively, these findings indicate that glucose metabolism differs between normal dogs and those with GRMD. In the case of GRMD canines, they exhibit elevated levels of resting insulin and glucose, along with a faster glucose uptake rate compared with that of typical canines. It appears that the increased presence of GLUT4 at the cellular membrane primes GRMD muscle to facilitate swift glucose uptake. Hence, glucose metabolism could potentially serve as biomarkers for evaluating disease progression and recovery following different therapies [76]. One example of this is the greater expression of glycogen synthase kinase 3 beta (GSK3B), a member of the insulin signaling pathway, in DMD mdx mice compared to a standard mouse [77].

The control of muscle protein synthesis in terms of nutrition is typically influenced by the insulin-dependent mechanism, where the release of insulin triggered by the intake of carbohydrates and/or proteins results in a nitrogen-conserving effect that facilitates a favorable net balance. However, it has been demonstrated that HMB supplementation does not elicit any impact on insulin supplementation [78]. Therefore, suppression of protein breakdown could be considered insulin-independent. Indeed, HMB has been shown to suppress ubiquitin-proteasomal regulated muscle protein breakdown and inhibition of myonuclear apoptosis by antagonizing mitochondrial-associated caspases [67]. Despite the inhibitory HMB effect on muscle protein breakdown, there were no obvious effects on the proteolytic markers such as *MURF1* and *MAFBX*, and therefore, how and through which proteolytic pathway HMB is regulating reductions in protein breakdown remains not clear [3,32]. Moreover, HMB has been shown to attenuate the increased phosphorylation of RNA-dependent protein kinase, which may activate *NF-κB*, whereas increased phosphorylation of mTOR could lead to phosphorylation and subsequent nuclear exclusion of Foxo3 [54]. HMB has been demonstrated to effectively prevent the nuclear accumulation of NF-κB and enhance the phosphorylation of mTOR in reaction to catabolic stimuli, as evidenced by Figure 4 [10]. Moreover, HMB has also been found to inhibit the expression of the Foxo3 gene in response to glucocorticoid-induced atrophy [54,66].

## 3. Common Genes

Data analysis carried out with the use of the Pathway Studio Mammal Plus software (V. 12.5.0.2, 2022), along with the analysis of literature data, allowed for the identification of aforementioned common pathways and additionally some genes of significant importance for the course of DMD disease and indicated as being under the influence of HMB (Figure 1). The main common genes are listed in (Table 1), along with their effect on DMD and HMB, and the literature reference. Those not mentioned above as taking part in common DMD/HMB pathways are discussed below.

One of the common genes is myostatin (*MSTN*), expressed in skeletal muscles and adipose tissues, which is a negative regulator of muscle growth [95], also known as growth and differentiation factor-8, the *TGF-*α superfamily member [96]. *MSTN* expression is greatly decreased, at both mRNA and serum protein levels, in patients affected by muscle wasting and atrophying diseases, including DMD [97]. It has been proven that *MSTN* is a promoter of muscle wasting having an impact on overall protein turnover by increasing protein degradation and inhibiting protein synthesis [11]. Wagner et al. (2002) [98] showed that deletion of *MSTN* attenuates the severity of muscular dystrophy. The positive effect of myostatin blockade on dystrophic mdx mice was also confirmed by Bogdanovich et al. (2002) [99].

In recent years, many attempts have been carried out to evaluate anti-myostatin therapy aimed to systemically abrogate myostatin/ActRIIB signaling in atrophying muscle tissue, which in turn may improve muscle function. Unfortunately, the conducted clinical trials using ex. RO7239361 and PF-06252616 were not successful [100,101,102]. Supplementation with HMB is known to be effective in preventing inflammation and myostatin-associated muscle atrophy [11]. Mobley et al. (2014) [103] proposed that the reversal of myostatin-induced atrophy in myotubes occurs via modulating Akirin-1/Mighty mRNA expression by HMB, L-leucine, and creatine monohydrate. Moreover, dietary HMB + β-ala can reduce the active form of myostatin in rats [104] and myostatin mRNA expression in pigs, exerting muscle growth-promoting effects [105]. Olveira et al. (2016) [106] mentioned a trend of lower plasma myostatin levels at 12 wk of HMB oral supplementation in patients with bronchiectasis. However, Wilson et al. (2012) [107] did not find any significant effect for myostatin after 10-week resistance training of rats supplemented with HMB. Despite the description of the positive effect of HMB on body composition and sensorimotor function during normal training and its impact on reducing muscle mass and strength loss in catabolic states, Park et al. (2013) [108] did not prove that myostatin as a factor responsible for the above-mentioned HMB-dependent changes.

Another gene, leptin (*LEP*), is secreted in white adipose tissue and its level depends on the amount of body fat [109]. It can activate several signal pathways such as JAK-STAT3, which are important for the regulation of energy homeostasis [110]; and PI3K, which regulates both food intake and glucose homeostasis [111]. In addition, MAPK, AMPK, and mTOR pathways have been proposed to be downstream of LEP [112]. Regarding DMD or BMD patients with risk factors such as obesity, hyperinsulinemia, and insulin resistance, *LEP* expression increases in individuals with age, white adipose tissue accumulation, and different degrees of metabolic syndrome [113]. The relative expression of this gene in DMD patients in the study by Söderpalm et al. (2007) [84] showed that they had increased serum *LEP* levels, which is probably due to the increased fat mass and treatment with glucocorticoids. *LEP* is one out of only a few proteins reported to be stably elevated in DMD [114]. Interestingly, a recent study demonstrated that HMB has a similar effect on *LEP* expression as was also observed in DMD disease. The administration of HMB during the prenatal period resulted in an indirect upregulation of leptin levels. This effect was achieved by augmenting the GH/IGF1 axis as well as the pituitary-gonadal axis, which in turn stimulated bone cellular proliferation and advanced collagen maturation [115].

Neuronal nitric oxide synthase (*nNOS*) is a protein that plays an important role in the production of the freely diffusible signaling molecule nitric oxide (NO). It has contributed to myofibrillar differentiation [116], modulation of contractile force [106], and exercise-induced glucose uptake in muscles [117]. It was demonstrated using a mouse experimental model that NO decreases vasoconstrictor response to activation of α-adrenergic receptors, and therefore has a critical role in the blood flow regulation in exercising skeletal muscle and also in DMD in case of which the lack of *nNOS* causes unopposed sympathetic vasoconstriction that can contribute to muscle fiber apoptosis or necrosis [118,119].

The lack of dystrophin results in the displacement and decrease of *nNOS* levels, which ultimately leads to elevated muscle fatigue. NO plays a key role in the S-nitrosylation and chromatin association of HDAC2, thereby causing an increase in activity levels in mdx mice [120]. The phenotype of iNOS KO mice became apparent from the third week, characterized by an increase in whole-body fat while lean mass was reduced, as well as liver weight [121]. The loss of nNOS in DMD appears to be a direct consequence of the deficiency of dystrophin [122]. Another investigation indicated that DMD can change *nNOS* expression. Guilbaud et al. (2018) [123] showed that *nNOS* expression is regulated by miR-31, miR-708, and miR-34c. Overexpression of these miRNA in DMD human myoblasts (DMDd45-52) leads to a decreased level of *nNOS*; accordingly, the inhibition of miR-708 and miR-34c can escalate *nNOS* expression [120,122,123,124]. Many studies have shown that HMB hurts *nNOS* expression [125,126]. The increased nitrate levels in macrophage cultures due to HMB exposure suggests that HMB may induce *nNOS* activity [90]. Based on this investigation, Wójcik et al., 2019 [127] suggested that similarly to other macrophage activators (such as LPS), HMB stimulates the activity of *nNOS*. However, Szcześniak et al. (2016) [128] studied the mechanism of action of HMB in equine satellite cells and demonstrated that *NOS2* (iNOS) gene expression may be activated by *MAPK14*. In their investigation, the *NOS2* expression declined. Similar results were obtained by other studies and showed that HMB also inhibited the LPS-induced *TNF-α*, *IL1β*, and *iNOS* expressions [127,129].

Lactate dehydrogenase (LDH) is one of the essential enzymes of the organism, and its abnormal activity is observed in many disorders [130,131]. Heinova et al. (1999) [132] showed significant variation between tissue distribution and activity of the enzyme and its isoenzyme patterns in the animal species. The extracellular level of LDH can be a marker for the detection of cell damage or cell death [133]. The increase in serum levels of CK, another enzyme and a biomarker of membrane fragility, muscle degeneration, and injury, was identified in individuals with DMD myopathy in more than half of the cases [134]. Skeletal muscle activity as well as the severity of the degenerative disease of the skeletal muscles leads to changes in CK and LDH levels. After muscle injury, the plasma level of the aforementioned CK and LDH is increasing [135,136,137]. Sadek et al.’s (2017) [93] study on DMD children showed that creatinine phosphokinase (CPK) in all cases was high and in 19 cases of 28 male DMD children, the LDH level was also elevated, in comparison to control individuals.

Another study on 96 malnourished DMD boys indicated a significant increase in muscle enzymes such as CK and LDH, as well as limb weakness [138]. Moreover, another investigation showed that the levels of LDH and CK were elevated for a 14-year-old Mongolian boy suffering from proximal muscle weakness [139]. The elevation of both enzymes was also proven in the mdx mouse model of DMD [140]. It is known that supplementation of HMB leads to decreased markers of muscle damage following mechanically strenuous exercise, including lower levels of LDH [141], CK [142,143], and muscular soreness [143]. In addition, HMB supplementation results in a decreased plasma CPK and LDH response after a prolonged run [141,144] and decreased CPK peak after a professional soccer game [145]. A similar effect was described in horses [137]. However, some investigations do not confirm the effect of HMB on the activity of CK and LDH [107,146,147].

## 4. Therapeutic Agents Used for DMD Treatment

### 4.1. Statins

Statins (HMG-CoA reductase inhibitors) are cholesterol-lowering drugs commonly used to manage and prevent cardiovascular and coronary heart diseases [148]. Statins are known to have various pleiotropic effects, including the enhancement of endothelial function, suppression of vascular inflammation and oxidation, and stabilization of atherosclerotic plaques [149]. The pharmacological activity of statins is based on their strong inhibition of the endogenous mevalonate pathway, which directly controls the biosynthesis of cholesterol. Through their interactions with HMG-CoA reductase, statins hinder the conversion of HMG-CoA to L-mevalonate, which ultimately inhibits the downstream biosynthesis of cholesterol. Lowering LDL cholesterol is the primary goal of statin treatment in humans. In muscular dystrophy, the rationale for using statin (simvastatin) is based on the additional cholesterol-independent, pleiotropic benefits of statins. These benefits include reducing oxidative stress, inflammation, and fibrosis, three key pathogenic processes in DMD that are major mediators of functional impairment observed in muscular dystrophy. Concerning oxidative stress, Whitehead et al. (2016) [150] found that NADPH oxidase 2 (NOX2) plays a significant role in the production of reactive oxygen species (ROS) in mdx mice, a mouse model of DMD with increased levels of NOX2 in mdx muscle and during stretched contractions. The ROS produced by NOX2 triggers the opening of stretch-activated channels (SACs), leading to excessive Ca^2+^ entry into dystrophic muscle fibers. This pathway results in high levels of ROS and Ca^2+^, which increase membrane permeability, and muscle damage, and reduce force production. However, the administration of simvastatin at different stages of the disease substantially improves general muscle health and functions [151].

The aforementioned observations depict that simvastatin has been efficacious in providing substantial care against muscle impairment as exhibited by the pronounced reductions in plasma creatine kinase, inflammation, fibrosis, and oxidative stress. Additionally, NOX2 levels also decreased to control values in simvastatin-treated mdx mice, and this was congruent with an amplified muscle force production. Of particular interest is the discovery that restoring dystrophin and/or the dystrophin-associated protein complex (DPC) is not essential to provide an improvement in dystrophic muscle health and force production. Simvastatin was found to not affect the expression of dystrophin homolog, utrophin, or other DPC proteins in mdx muscle. Additionally, the increase in specific muscle force brought about by simvastatin was found to be comparable to that of the most effective mini-dystrophin gene therapy construct [152].

In recent years, the therapeutic function of statins has been directed towards the stimulation of autophagy, which plays a crucial role in preserving cellular homeostasis and is responsible for the elimination of unfavorable cells or abnormal organelles within cells. The malfunctioning of the autophagy pathway has been attributed to the etiology of various disorders, including muscular dystrophies. De Palma et al. (2012) [153] showcased that muscles from patients suffering from DMD and mdx mice exhibit severe impairment of autophagy. The imbalance of autophagy is causative of muscle degeneration and is accompanied by hyperactivation of AKT as a result of muscle-specific deletion of the mTOR. This process leads to the inhibition of autophagy and a muscle phenotype similar to that observed in muscular dystrophy. Statin-induced autophagy leads to the normalization of AKT and mTOR signaling [154]. The declining level of the NOX2 pathway may ameliorate autophagy [155].

However, some case reports are indicating that the use of statins, especially in conjunction with other drugs (fibrates), leads to some side effects. Mendes et al. (2014) [156] demonstrated that statins, widely prescribed for the reduction of *LDL* cholesterol, may develop rhabdomyolysis, a syndrome characterized by muscle necrosis and release of intracellular muscle contents into systemic circulation. The manifestation of rhabdomyolysis in patients is characterized by indescribable muscle aches and weakness. The diagnosis of rhabdomyolysis is confirmed by an increase in serum CK and the presence of myoglobinuria. The identification of individuals with an amplified likelihood of statin-induced rhabdomyolysis is of growing importance, as it will allow for the prevention of potential complications and further improve the positive benefit-risk ratio of statins.

Another well-known side effect of statin therapy is myopathy. A rare variant of this disease is statin-induced necrotizing autoimmune myopathy characterized by muscle weakness and marked persistent elevation of creatine phosphokinase, despite statin discontinuation. Diagnosis is verified through the presence of anti-HMG-CoA reductase autoantibody [157]; and the administration of steroids, followed by intravenous immunoglobulin treatment, gradually ameliorates the patient’s muscle weakness, CPK, and liver functions. The protective role of *IGF1* has been studied in patients with florid Cushing’s syndrome [158]. Individuals with low *IGF1* after curative surgery for Cushing’s syndrome have an adverse long-term outcome of hypercorticism-induced myopathy. In turn, Kajstura et al. (2001) [159] demonstrated that in diabetic patients, *IGF1* has been found to protect the myocardium against the unfavorable consequences of diabetes through its ability to decrease the synthesis of Ang II, thereby resulting in a reduction of reactive damage and consequent cell death. The overexpression of *IGF1* has been observed to inhibit the progression of diabetic cardiomyopathy and oxidative stress related to angiotensin II.

**Figure 5:**
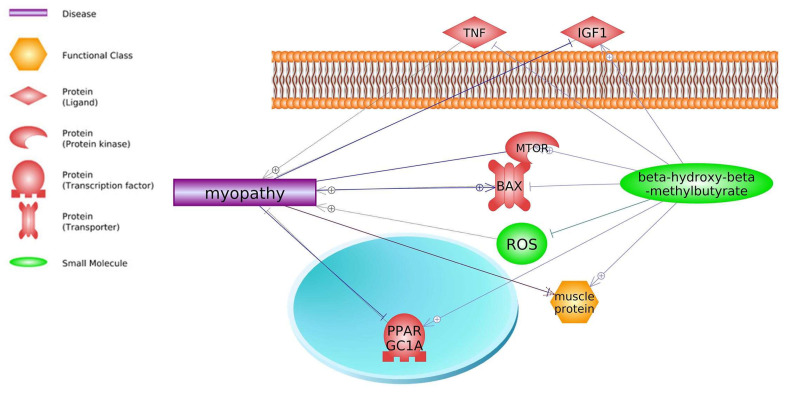
Myopathy-related genes and their interactions with HMB (based on Pathway Studio Web Mammalian). Tumor Necrosis Factor (TNF), Insulin-Like Growth Factor 1 (IGF1), BCL2 Associated X, Apoptosis Regulator (BAX), PPARG Coactivator 1 Alpha (PPARGC1A), Reactive Oxygen Species (ROS), Mechanistic Target of Rapamycin (mTOR). The “+” symbol at the end of the arrow corresponds to stimulation, the “┤” symbol corresponds to inhibition.

**Figure 6:**
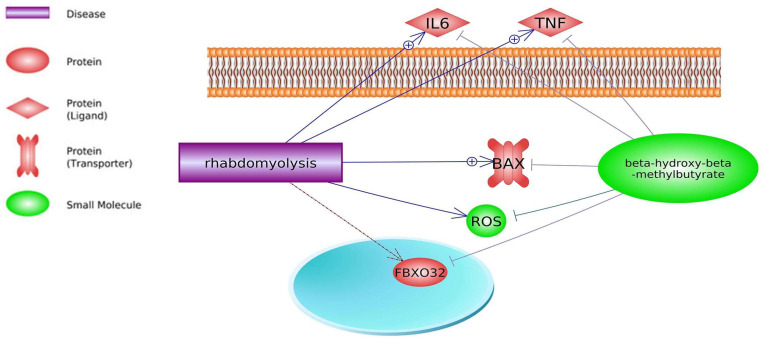
Rhabdomyolysis-related genes and their interactions with HMB (based on Pathway Studio Web Mammalian). F-Box Protein 32 (FBXO32), Tumor Necrosis Factor (TNF), Interleukin 6 (IL6), BCL2 Associated X, Apoptosis Regulator (BAX), Reactive Oxygen Species (ROS). The “+” symbol at the end of the arrow corresponds to stimulation, the “┤” symbol corresponds to inhibition.

HMB associated with low-intensity exercise improves skeletal muscle regeneration through the IGF pathway and greater AKT phosphorylation in rats [160]. HMB also decreases fibrosis during the regeneration process, mainly due to significantly decreased *TGFβ1* levels. *TGFβ1* inhibition leads to muscle regeneration by reducing fibrosis. Therefore, HMB supplementation accelerates connective tissue repair of injured muscles. Some proteins such as TNF [161], BAX [162], and ROS [163] can enhance myopathy of skeletal muscle. Studies of Bongiovanni et al. (2020) [164] have shown that both acute and chronic supplementation of HMB before exercise might improve functional and biochemical markers of exercise-induced muscle damage. HMB’s ability to protect against muscle loss may be due in part to putative inhibition of myonuclear apoptosis via regulation of mitochondrial-associated caspase signaling [67]. HMB as a dietary supplement can reduce statins’ adverse effects. The literature analysis by Pathways Studio indicates that HMB may indirectly ameliorate statin drawbacks such as myopathy and rhabdomyolysis (Figure 5 and Figure 6; keywords: myopathy, rhabdomyolysis, and β-hydroxy-β-methylbutyrate).

### 4.2. Glucocorticoids

Corticosteroids have become a widely used and effective treatment for various inflammatory and autoimmune diseases. They are used as replacement therapy in adrenal insufficiency at physiological doses, as well as in therapeutic doses for the management of dermatologic, ophthalmologic, rheumatologic, pulmonary, hematologic, and gastrointestinal disorders. These synthetic analogs of natural steroid hormones produced by the adrenal cortex possess glucocorticoid and/or mineralocorticoid properties. Glucocorticoids are mainly responsible for carbohydrate, fat, and protein metabolism, and have anti-inflammatory, immunosuppressive, anti-proliferative, and vasoconstrictive properties [165,166].

The use of corticosteroids in patients with DMD recommends prednisone or deflazacort for short-term benefit in muscle strength and function, according to guidelines [167]. These corticosteroids possibly increase survival over 5–15 years of treatment, improve strength, improve timed motor function, and delay the age at loss of ambulation. They also slow the development of scoliosis and reduce the need for scoliosis surgery. Finally, they delay the onset of cardiomyopathy. The molecular basis of the beneficial effect of glucocorticoids is not understood. These hormones have diverse impacts on muscle metabolism, growth, and development. Although some of these impacts are destructive in nature, such as an increase in protease activity, there have been observed positive stimulatory effects of glucocorticoids on myoblast proliferation and differentiation [168].

In human muscle cultures, there is a gradual loss of myotubes and decrease in dystrophin content. However, these adverse effects can be prevented by administering glucocorticoid treatment to the muscle cultures. In contrast to routine cultures, the content of dystrophin homolog, utrophin in dexamethasone (DEX)-treated DMD myotube cultures is significantly greater than in the corresponding untreated cultures [169]. In mdx mice, deflazacort treatment promotes functional gains, myogenic differentiation, myoblast fusion, and laminin expression in regenerating dystrophic muscle [170], stabilization of muscle fiber membranes [171], and differential regulation of genes in muscle fibers [172]. All that action leads to slower DMD progression and improvement of muscle strength [173]. Moreover, glucocorticoids may induce weakness and atrophy of muscles in DMD because they act directly on myofibers, decline protein synthesis, and increase the rate of protein catabolism, as well as stimulate the catabolic AKT1/FOXO1 pathway [174].

Jensen et al. (2017) [175] used human muscle biopsies to study the muscular response of deflazacort treatment in boys with DMD. The evaluation encompassed scrutiny of the mRNA transcripts responsible for the activation of satellite cells, myogenesis, regeneration, adipogenesis, muscle growth, and tissue inflammation. Transcript levels for *ADIPOQ*, *CD68*, *CDH15*, *FGF2*, *IGF1R*, *MYF5*, *MYF6*, *MYH8*, *MYOD*, *PAX7*, and *TNF-α* were significantly different in treated patients vs. normal muscle. In addition, deflazacort treatment reduced CK activity in DMD patients but there was no effect on muscle protein expression.

While the advantages of corticosteroids in DMD are becoming more evident, there still exists uncertainty regarding the equilibrium between benefits and side effects that arise with prolonged treatment. As individuals with DMD advance past the point of losing ambulation, the relative significance of side effects that were previously well-tolerated is elevated, and management becomes more intricate. In non-ambulatory patients with active declines in heart and lung function, side effects such as weight gain, hypertension, glucose intolerance, and their effects on growth and bone density are observed [176]. One of the most frequent side effects of using corticosteroids in kids is weight gain [177]. Moreover, gradually DMD children are getting Cushing’s syndrome expressed by excessive storage of adipose tissue in their face, trunk, and upper back [165]. Osteoporosis is another adverse effect related to steroids. The risk of bone fracture results from low calcium absorption, accelerated bone catabolism, and decreased bone formation [165]. However, data are suggesting that osteoporosis initiation is not linked with steroid treatment. Corticosteroids and methotrexate may increase the risk of *GH* deficiency and lower bone mineral density [178]. Moreover, corticosteroids directly affect *GH* secretion and suppress the production of *IGF1,* as well as interfere with bone metabolism [179].

Corticosteroid therapy often decreases serum magnesium levels and induces muscle atrophy in patients. Zheng et al. (2021) [52] demonstrated in rats (both in vivo and in vitro) that Mg^2+^ supplementation reversed the corticosteroid-induced lower diameter of myotubes and activated *MuRF1* and *MAFbx* expression, thus providing evidence that Mg^2+^ protected muscle from atrophy by inhibiting muscle protein degradation.

**Figure 7:**
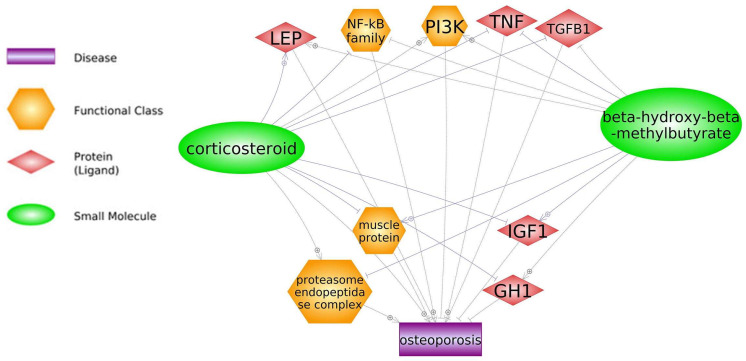
Corticosteroids-related genes and their interactions with HMB (based on Pathway Studio Web Mammalian). Insulin-like Growth Factor (IGF), Leptin (LEP), Phosphoinositide 3-kinases (PI3K), Nuclear Factor Kappa B Subunit (NF-κB), Tumor Necrosis Factor (TNF), Transforming Growth Factor-Beta 1 (TGFB1), Growth Hormone (GH). The “+” symbol at the end of the arrow corresponds to stimulation, the “┤” symbol corresponds to inhibition.

HMB supplementation can diminish corticosteroid side effects such as osteoporosis development through *IGF-1*, *GH*, and muscle protein regulation (Figure 7; keywords: corticosteroid, β-hydroxy-β-methylbutyrate). HMB, a metabolite of leucine, has been acknowledged for its anabolic properties concerning muscle proteins and ability to hinder protein degradation. HMB has been administered to patients afflicted with muscle atrophy arising from debilitating illnesses like AIDS, cancer, and chronic obstructive pulmonary disease (COPD) [180]. Increasing muscle protein synthesis leads to the suppression of adverse effects of osteoporosis and corticosteroids [181]. The inhibitory effects of HMB on the NF-κB pathway are achieved by diminishing *IL17* expression. This, in turn, leads to a decrease in the degradation of muscle cells. By acting on downstream genes of the NF-κB pathway—particularly *IL6*, *TNF-α*, and *IL1β*—HMB deters apoptosis and enhances myoblast proliferation. Consequently, the regulation exerted by HMB on NF-κB signaling aids in repairing myoblast injuries and promotes the overall recuperation process [182]. In addition, in pigs challenged with LPS, the addition of dietary HMB led to a significant decrease in the mRNA expression of *MyD88* and *NF-Κb*, *p65* (*p* < 0.05). This suggests that HMB may exert a modulating effect on the NF-κB pathway [105].

HMB exerts protective effects against DEX-induced muscle atrophy [183]. In vivo, experiments performed on rats demonstrated that co-administration of HMB attenuates the loss of lean mass and shrinkage of the fibers and restores muscle functionality. In an in vitro model utilizing L6 myotubes, the prevention of muscle atrophy through HMB was achieved through the modulation of the autophagy-lysosomal system. The normalization of autophagy through HMB was largely dependent upon the phosphorylation of FOXO3A, a consequence of an increase in P13K/AKT signaling in the cytosol [183]. Studies conducted both in vivo and in vitro suggest that HMB has the potential to protect against muscle atrophy followingDEX treatment by regulating the AKT/FOXO axis, which controls autophagy and ubiquitin proteolysis.

## 5. Conclusions

**Figure 8:**
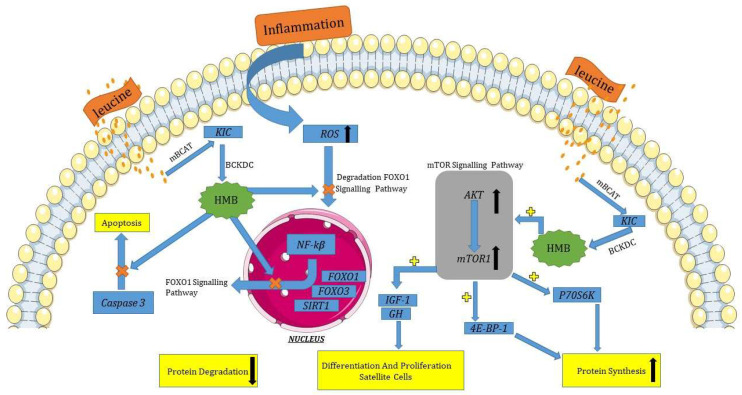
Summary of the interactions of signaling pathways related to the development of DMD and mechanism of action of HMB in skeletal muscle cell (prepared based on Landi et al., 2019 [184]). Reactive Oxygen Species (ROS), Ketoisocaproate (KIC), Protein Kinase B (AKT), Mechanistic Target of Rapamycin (mTOR), Branched-Chain Ketoacid Dehydrogenase Complex (BCKDC), p70 Ribosomal S6 Kinase (P70S6K), Eukaryotic Translation Initiation Factor 4E-Binding Protein 1 (4E-BP-1), Insulin-like Growth Factor 1 (IGF1), Growth Hormone (GH), Forkhead Box O1 (FOXO1), Forkhead Box O3 (FOXO3), Sirtuin 1 (SIRT1), Nuclear Factor Kappa B (NF-κB), Mitochondrial Branched-Chain Amino Acid Transaminase (mBCAT ). The “+” symbol corresponds to stimulation, the “x” symbol to inhibition. Black arrows pointing up and down indicate an increase or decrease in protein activity or process intensity, respectively.

Based on the literature, Pathway Studio analysis, common genes, and pathways have been found for both HMB and DMD. Figure 8 provides a summary of the interactions of the above-described signaling pathways related to the development of DMD and mechanism of action of HMB. In this article, we demonstrate that HMB appears to be a potential supplement for DMD patients. As a consequence, limited use of statin and corticosteroids may eliminate the harmful effects of these drugs. In recent times, gene-focused techniques have been formulated to create a truncated dystrophin protein in DMD muscles. These procedures include the use of ‘exon skipping’ oligonucleotides, which are currently undergoing clinical trials. Nonetheless, despite the conceptual appeal of these approaches, several regulatory and technical hurdles must be overcome before these novel methods can be accessible to DMD patients. Furthermore, in the case of exon skipping, each oligonucleotide is tailored to target a specific dystrophin mutation, thus necessitating independent testing on a specific group of patients with that mutation. This will further delay the time required to access a potential treatment and may make room for the use of therapy-enhancing supplements such as HMB.

## Figures and Tables

**Table 1:** The main genes common for DMD and HMB.

Gene	DMD Effect	References	HMB Effect	References
*MSTN*	inhibitor	Chiu, W. et al. (2020) [81]	stimulator	Shirvani, H. et al. (2020) [82]
*AKT1*	stimulator	Peter, A.K.; Crosbie, R.H. (2006) [47], Kornegay, J.N. et al. (2012) [83]	stimulator	Salto, R. et al. (2015) [49] Schnuck, J.K. et al. (2016) [79]
*LEP*	stimulator	Söderpalm, A.C. et al. (2007) [84]	stimulator	Świetlicka, I. et al. (2021) [85]
*IL6*	stimulator	Rodríguez-Cruz, M. et al. (2018) [17] Gallardo, F.S. et al. (2021) [60]	inhibitor	Miyake, S. et al. (2019) [58]
*CASP3*	positive effect	Parrotta, E.I. et al. (2020) [63]	inhibitor	Eley, H.L. et al. (2008) [10] Hao, Y. et al. (2011) [67]
*MAPK (MAPK14)*	biomarker	Cotán, D. et al. (2016) [86]	inhibitor	Eley, H.L. et al. (2008) [10]
*MyoD family*	unknown	Gonçalves, M.A.F.V. et al. (2008 and 2011) [87,88]	stimulator	Kim, J.-S. et al. (2013) [46]
*nNOS*	unknown	Tian, L.J. et al. (2014) [89]	stimulator	Peterson, A.L. et al. (1999) [90]
*NF-κB*	stimulator	Wattin, M. et al. (2018) [53] Finkel, R.S. et al. (2021) [91]	inhibitor	Smith, H.J. et al. (2005) [4] Miyake, S. et al. (2019) [58]
*LDH*	stimulator	Luce, L.N. et al. (2016) [92] Sadek, A.A. et al. (2017) [93]	unknown	Tsuchiya, Y. et al. (2019) [94]

DMD—Duchenne muscular dystrophy; HMB—β-hydroxy-β-methylbutyrate acid; Myostatin (*MSTN*), AKT (*AKT1*), Serine/Threonine Kinase 1, Leptin (*LEP*), Interleukin 6 (*IL6*), Caspase 3 (*CASP3*), Mitogen-Activated Protein Kinase (*MAPK*), Myogenic Differentiation (*MyoD*), Nitric Oxide Synthase (*nNOS*), Nuclear Factor Kappa B Subunit (*NF-κB*), Lactate Dehydrogenase (*LDH*).
